# COVID-19 Preventive Practices, Parent–Child Conflict, and Depressive Symptoms Among Chinese Adolescents

**DOI:** 10.3390/bs16091654

**Published:** 2026-09-15

**Authors:** Tong Zhou, Luyan Xu, Xiaohua Bian, Luhao Wei, Junsheng Liu

**Affiliations:** 1 School of Psychology, Shanghai Normal University, Shanghai 200234, China; zhoutong@shnu.edu.cn; 2 School of Psychology and Cognitive Science, East China Normal University, Shanghai 200062, China; 18762349989@163.com; 3 Suzhou No.1 High School, Suzhou 215005, China; 4 School of Educational Science, Zhengzhou Normal University, Zhengzhou 450044, China; bxh1999@zznu.edu.cn; 5 Department of Psychology, University of Virginia, Charlottesville, VA 22901, USA; ykt9dq@virginia.edu

**Keywords:** adolescence, preventive practices, depressive symptoms, parent–child relations, gender differences, COVID-19

## Abstract

The COVID-19 pandemic affected adolescent mental health. However, the concurrent associations among adolescents’ preventive practices, parent–child conflict, and depressive symptoms during the initial lockdown remain unclear. This cross-sectional study included 2558 adolescents (48.75% girls; M age = 13.06 years) from Zhengzhou, China, of whom 2543 were included in the analytic sample after cases missing age or class identifiers were excluded. All focal constructs were assessed once by adolescent self-report. Latent moderated mediation analyses showed that preventive practices were directly and negatively associated with depressive symptoms. A significant indirect association was observed through parent–child conflict such that greater engagement in preventive practices co-occurred with less parent–child conflict and fewer depressive symptoms. The negative direct association between preventive practices and depressive symptoms was stronger for boys, whereas the positive association between parent–child conflict and depressive symptoms was stronger for girls. However, these interaction terms added only ΔR^2^ = 0.003 and 0.004 in observed-score sensitivity models and were not statistically significant in the subsample sensitivity analysis. These findings identify parent–child conflict as a concurrent correlate of the association between preventive practices and depressive symptoms during the pandemic.

## 1. Introduction

The outbreak of the coronavirus disease 2019 (COVID-19) disrupted adolescents’ schooling, face-to-face peer contact, daily routines, and family life worldwide. The Ministry of Education issued a nationwide school closure policy from the end of January to May in 2020, and more than 180 million Chinese primary and secondary school students began their online learning at home (UNICEF China, 2020). Longitudinal evidence indicates that depressive symptoms increased on average from before to during the pandemic, but the magnitude and direction of change varied across samples, periods, and social contexts (Madigan et al., 2023; Wolf & Schmitz, 2024). Reviews further identify multiple correlates of adolescent adjustment, including pre-existing mental health, social isolation, school disruption, parental mental health, family functioning, and access to support (Branje & Morris, 2021; Wolf & Schmitz, 2024). These findings underscore the importance of identifying the behavioral and family factors associated with individual differences in adolescents’ depressive symptoms during the pandemic.

Preventive practices such as wearing facial masks and disinfection (e.g., handwashing) have been key to reducing COVID-19 morbidity and mortality (Scholz & Freund, 2021). In terms of mental health, preventive practices were found to be related to reduced risk of mental health problems among adults across different countries, including the United States (C. H. Liu et al., 2022), China (C. Wang et al., 2020), and Indonesia (Sitanggang et al., 2021). Notably, a recent systematic review and meta-analysis revealed that, compared with adults, adolescents showed poorer engagement in COVID-19 preventive practices than adults, where boys showed a lower behavioral compliance than girls (Li et al., 2022). These findings suggest that greater attention to adolescents’ preventive practices and their psychological implications are needed to inform future preventive efforts. In addition, due to the COVID-19 home confinement mandate, parent–child interactions became an even more significant social source of support and stress for adolescents. Parental socialization can play a key role in adolescents’ endorsement of preventive practices (Ren et al., 2021). Nevertheless, preventive practices, parent–child conflict, and depressive symptoms have rarely been examined together within the same adolescent model. The present study addressed this issue by examining the association between adolescents’ preventive practices and depressive symptoms, the indirect association through parent–child conflict, and whether these associations differed by gender.

### 1.1. Preventive Practices and Depressive Symptoms

Preventive practices during the COVID-19 pandemic refer to protective behaviors against the spread of the virus, such as handwashing, wearing face masks, and maintaining physical distance (Ren et al., 2021; Scholz & Freund, 2021), which were found to reduce viral transmission and infection rates (Scholz & Freund, 2021). In addition, several studies found that higher engagement in preventive practices was associated with better psychological adjustment in adults during the COVID-19 pandemic (Aydin & Kaya, 2021; C. H. Liu et al., 2022; Yuan et al., 2022). For instance, Yuan et al. (2022) found that the decreased wearing of masks in the workplace was associated with workers’ poorer sleep quality three months later. Among U.S. adults, preventive practices during the COVID-19 pandemic were found to protect those with higher levels of daily hassles from moderate levels of depressive symptoms (C. H. Liu et al., 2022). Greater compliance with preventive health behaviors was associated with lower levels of depression, anxiety, and stress among Turkish adults (Aydin & Kaya, 2021). Researchers applied stress and coping theory (Lazarus & Folkman, 1984) and self-efficacy theory (Bandura, 1977) to interpret such findings. Specifically, preventive practices may function as situation-specific coping responses, as the active steps taken to meet the pandemic-specific situational demands may, in turn, ameliorate the effects of the stressor and lead to adaptive outcomes in stressful encounters (Cheng & Cheung, 2005). Moreover, endorsement of preventive practices was also interpreted as one’s perceived ability to cope with stressful situations, which may increase individuals’ perceived control and therefore be associated with better mental health (Sitanggang et al., 2021).

Compared with adults, adolescents were found to endorse fewer preventive practices (Li et al., 2022). For instance, a meta-analysis of 23 studies confirmed the poorer practice of COVID-19 preventive behaviors among children and adolescents compared with adults (Li et al., 2022). Although previous research has identified factors associated with adolescents’ engagement in preventive behaviors (Park & Oh, 2022), comparatively fewer studies have examined their psychological correlates. Available evidence suggests that preventive practices, including face-mask use, were associated with lower levels of COVID-19-related anxiety among children and adolescents (Burak, 2023; Sharma et al., 2020). For instance, based on local policies and suggestions made by world health agencies (e.g., the WHO), researchers in Nepal reasoned that one of the protective factors for youth’s mental health during the COVID-19 outbreak was the use of face masks (Sharma et al., 2020). In empirical studies, seeing COVID-19 cases at school was identified as a risk factor for COVID-19 anxiety in Turkish children, while children’s preventive practices were associated with lower levels of COVID-19 anxiety in both social and individual domains (Burak, 2023). However, these studies did not consider depressive symptoms together with adolescents’ family relationships. It therefore remains unclear how adolescents’ preventive practices were concurrently associated with depressive symptoms and parent–child conflict during the initial lockdown. Guided by stress and coping and self-efficacy perspectives (Bandura, 1977; Lazarus & Folkman, 1984) and the existing evidence among adults, we predicted that adolescents’ preventive practices would be negatively associated with their depressive symptoms in the context of the COVID-19 pandemic. Further, to understand potential family process correlates of this association among Chinese adolescents, we examined parent–child conflict.

### 1.2. The Indirect Association Through Parent–Child Conflict

As the primary socialization agent, parents are responsible for teaching and monitoring their children’s behaviors, which include adherence to recommended health practices that protect children’s health during the COVID-19 pandemic (Peplak et al., 2021; Ren et al., 2021). The existing literature shows that, during the COVID-19 lockdown, parent–child conversations centered around personal and social hygiene topics more than in the period before the pandemic (Tambling et al., 2021). Moreover, parents introduced more than a dozen novel COVID-19-related rules in family routines during the COVID-19 lockdown (Bülow et al., 2021). Self-determination theory suggests that adolescents are more likely to internalize parent-endorsed rules when parents acknowledge their perspectives, provide meaningful rationales, and support volitional functioning, whereas controlling communication may elicit resistance (Ntoumanis et al., 2021; Soenens et al., 2007). Social-domain theory similarly proposes that adolescents’ acceptance of parental rules depends partly on whether parental authority is viewed as legitimate within the relevant domain (Smetana & Asquith, 1994; Smetana & Rote, 2019). Consistent with this view, Bülow et al. (2021) found that, after the COVID-19 lockdown, most adolescents in the Netherlands perceived COVID-19-related family rules as legitimate, and those who thought that the rules were more legitimate showed less oppositional behavior toward their parents than their peers who perceived these rules as less legitimate. Accordingly, adolescents who accept and internalize parent-endorsed health guidance may show both greater engagement in preventive practices and less opposition toward their parents. Although autonomy support and rule legitimacy were not assessed directly, the literature provides a family process basis for expecting adolescents’ preventive practices to be associated with less parent–child conflict.

In support of this notion, prior findings have suggested an association between the parent–child relationship and adolescents’ health behaviors. For example, close bonds and positive interactions in supportive family relationships were associated with adolescents’ compliance with parent-encouraged health behaviors (Franko et al., 2008). During the COVID-19 pandemic, lower compliance with requests for family distancing was related to greater anger and more conflict between family members (Jeon et al., 2022). We therefore expected adolescents’ compliance with parental guidance and implementation of preventive practices to be associated with less parent–child conflict during the COVID-19 pandemic.

Parent–child conflict has consistently been associated with children’s and adolescents’ psychological problems (L. Wang et al., 2021). A recent three-level meta-analysis of 46 studies likewise documented a moderate positive association between parent–adolescent conflict and depressive mood while also showing that the association was larger in cross-sectional than longitudinal studies (Y. Wang & Tang, 2025). Based on family functioning theory, impaired family functioning, including heightened parent–child conflict, may be detrimental to adolescents’ psychological adjustment (Qu et al., 2021). Adolescents who perceived more parent–child conflict during COVID-19 home quarantine also reported more depressive symptoms (Rogers et al., 2021). Based on these findings, we expected adolescents’ preventive practices to be concurrently associated with less parent–child conflict and fewer depressive symptoms.

### 1.3. The Moderating Role of Gender

Previous research has primarily documented gender differences in the levels of preventive practices. During the COVID-19 pandemic, girls and women generally reported greater engagement in practices such as handwashing, disinfecting frequently used items, and wearing face masks than boys and men (Cvetković et al., 2020). Among adolescents, girls similarly reported greater compliance with preventive guidance, better COVID-19-related health literacy and attitudes, and more frequent engagement in preventive practices than boys (Riiser et al., 2020; Saurabh & Ranjan, 2020).

These mean-level differences suggest that gender may be relevant to adolescents’ preventive practices. Gender socialization perspectives suggest that boys and girls may differ in how they respond to parent-endorsed rules and experience family discord (Hill & Lynch, 1983; Shek, 2002). However, the available empirical evidence does not provide a consistent basis for predicting which particular association, or the overall indirect association, would be stronger for either boys or girls. We therefore examined gender as a potential moderator of the association between preventive practices and parent–child conflict and indirect association through parent–child conflict on an exploratory basis.

### 1.4. The Current Study

**Figure 1:**
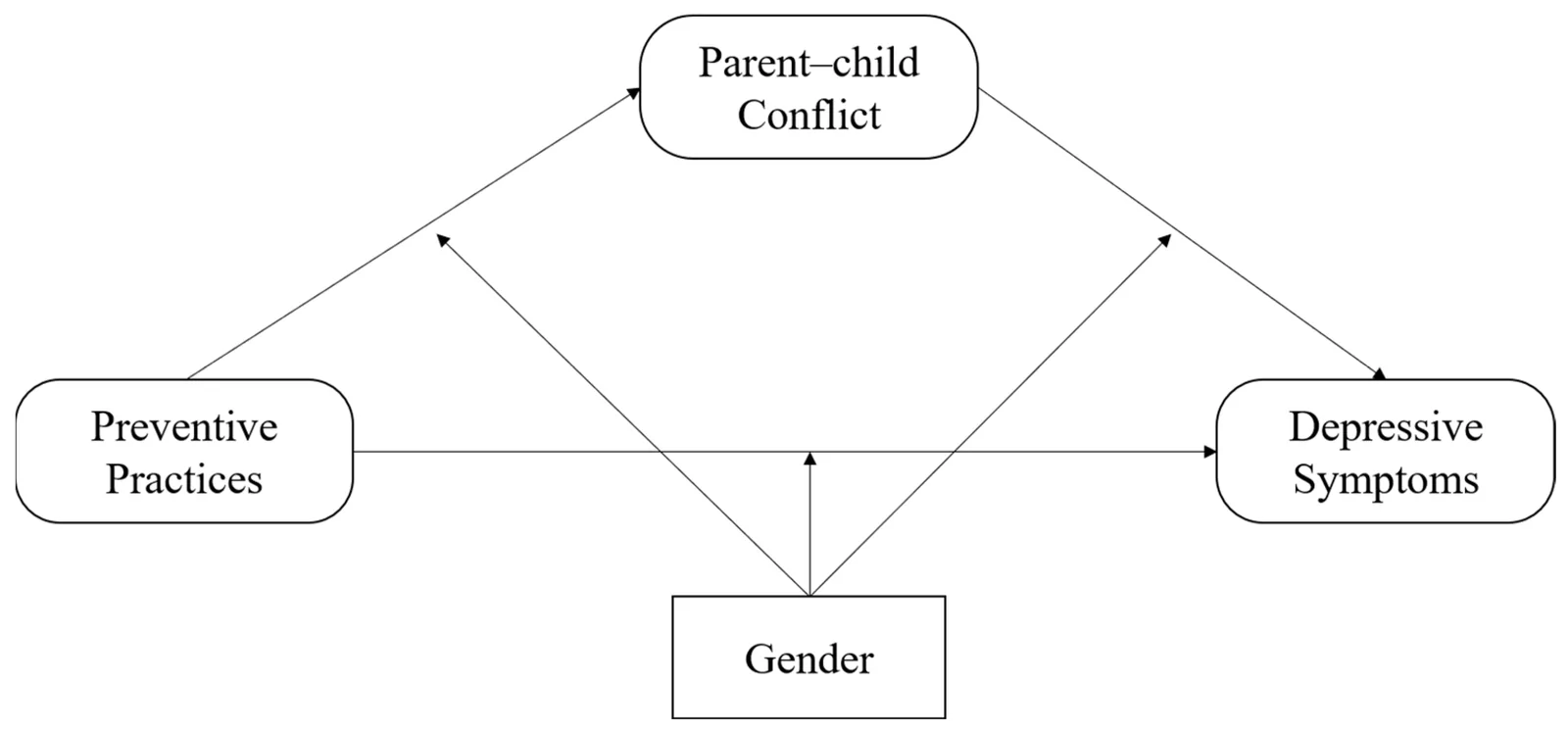
Conceptual framework of the associations among gender, preventive practices, parent–child conflict, and depressive symptoms. Note: Covariates include age, COVID-19-related events among relatives or close friends, COVID-19-related events in the community, and household socioeconomic status (SES).

Based on the limited available empirical and theoretical evidence, the current study proposed a moderated mediation model to examine the indirect association through parent–child conflict in the association between adolescents’ preventive practices and depressive symptoms. In addition, the moderating role of gender in the overall model was also explored. Previous studies have linked adolescent age, household socioeconomic status (SES), and living conditions during the outbreak of COVID-19 with adolescents’ mental health (J. Liu et al., 2021). Thus, these variables were included as potential covariates in the proposed model (see Figure 1).

Specifically, preventive practices are hypothesized to be negatively correlated with depressive symptoms in adolescents (Hypothesis 1). Parent–child conflict was expected to carry a significant indirect association between adolescents’ preventive practices and depressive symptoms (Hypothesis 2). Given the limited and potentially divergent evidence concerning gender differences across the component paths, no directional gender hypothesis was specified.

## 2. Materials and Methods

### 2.1. Participants

The present sample was drawn from the same broader COVID-19 family project as J. Liu et al. (2021). Of the 2558 adolescents analyzed here, 1594 were also analyzed, together with their mothers, in the study of J. Liu et al. (2021). The earlier article examined maternal reports of daily routines and adolescent reports of parent–child conflict, loneliness, and depressive symptoms. It did not analyze preventive practices, which are the focal predictor in the present study. In the present study, parent-reported information sufficient to construct household SES was available for 1946 adolescents. This number exceeds the 1594 overlapping mother–adolescent dyads because household SES information could be provided by either the adolescent’s mother or father, whereas J. Liu et al. (2021) included only adolescents with linked maternal data.

Participants in the present study were drawn from 80 classes across four public schools in Zhengzhou, the capital city of Henan province in central China (48.75% girls, M age = 13.06 years, SD = 1.56 years; age range = 10–16 years). During the outbreak of COVID-19, Henan province, which borders Hubei province, where Wuhan is located, was one of the provinces with the strictest measures and restrictions in China. Furthermore, 75% of responding parents reported monthly incomes below RMB 5000 (approximately US$707, based on the April 2020 average exchange rate of RMB 7.0708 per US$1), and 13.4% reported having a bachelor’s degree or higher. For context, official statistics for Zhengzhou in 2020 reported average monthly wages of approximately RMB 4672 among employees in urban private units and RMB 7250 among employees in urban non-private units, while 28.99% of residents had attained college-level education (Zhengzhou Municipal Bureau of Statistics, 2021a, 2021b). These comparisons suggest that the sample may have been somewhat less socioeconomically advantaged than the broader Zhengzhou population, particularly in parental education. Additionally, 12% of adolescents reported COVID-19-related events in their communities, and 1.05% reported exposure among relatives or close friends.

### 2.2. Procedure

Data were collected in April 2020, when Chinese schools were still under closure policies implemented by the Ministry of Education, and students were mandated to remain at home and study online. A Chinese online platform (Wenjuanxing) was used to collect the data for this research study, and the survey followed the CHERRIES guideline (Eysenbach, 2004). Due to the COVID-19 school closure, online class message groups were organized by head teachers. With the assistance of the school principals and head teachers, research assistants distributed information about the study and electronic consent forms to parents through these groups. Before participation, parents were informed of the study purpose, procedures, voluntary nature of participation, confidentiality protections, and their right to withdraw without penalty. Electronic informed consent was obtained from the parents of all participating adolescents. The adolescents were also provided with an age-appropriate explanation of the study and gave electronic assent before completing the survey. Only families for whom both parental consent and adolescent assent had been obtained were permitted to proceed with the survey. Parents completed a brief questionnaire assessing family demographic characteristics, such as monthly household income, after which their children completed the adolescent questionnaires described below. The study procedures were approved by the Research Ethics Review Committee at East China Normal University.

### 2.3. Measures

#### 2.3.1. Depressive Symptoms

Adolescents reported their depressive symptoms using the Chinese version of the Center for Epidemiologic Studies Depression Scale (CES-D; Radloff, 1977). The CES-D has demonstrated satisfactory psychometric properties among mainland Chinese adolescents (M. Wang et al., 2013). The scale consists of 20 items (e.g., “I felt down and unhappy”) rated from 0 (not at all) to 3 (a lot). Items are rated on a 4-point Likert scale ranging from 0 = not at all to 3 = a lot. After the four positively worded items were reverse-scored, item scores were summed to produce the conventional total score. A total of 638 adolescents (24.94%) scored at or above the screening cutoff of 16 (Radloff, 1977), indicating elevated depressive symptoms rather than a clinical diagnosis. This measure has been used in Chinese child and adolescent samples during the COVID-19 pandemic (J. Liu et al., 2021). The exact items used in the current study can be found in Appendix A. Confirmatory factor analysis supported a good model fit (CFI = 0.92, TLI = 0.91, RMSEA = 0.05, SRMR = 0.05), and the standardized loadings ranged from 0.21 to 0.82. The lowest loading was observed for Item 4, a positively worded and reverse-scored item^1^. Internal reliability was α = 0.82, and McDonald’s Ω was 0.88 in the current sample.

#### 2.3.2. Preventive Practices

We used a 3-item measure to assess adolescents’ preventive practices during the COVID-19 quarantine period. Adolescents self-reported engagement in/compliance with the following preventive practices: (a) “pay attention to disease preventive guidelines;” (b) “wear a facial mask or engage in other necessary preventive actions when they go out;” and (c) “wash their hands more often.” Each item was rated on a 5-point Likert scale (1 = not at all to 5 = very much). Mean scores were calculated to represent adolescents’ participation in preventive practices during COVID-19. This measure has been used among Chinese children and adolescents during the COVID-19 outbreak (Ren et al., 2021). Because the three-item one-factor model was just-identified, global model fit indices were not interpreted. The standardized factor loadings were 0.61, 0.74, and 0.78. Internal reliability was α = 0.74, and McDonald’s Ω was 0.75 in the current sample.

#### 2.3.3. Parent–Child Conflict

Adolescents reported on the level of parent–child conflict during the COVID-19 quarantine using the adapted conflict scale of the Parental Environment Questionnaire (PEQ) (Elkins et al., 1997). The original PEQ Conflict subscale comprises 12 items. For the present study, we used a 7-item version previously adapted for a Chinese adolescent sample during the COVID-19 pandemic (J. Liu et al., 2021). The adaptation was conducted by the research team for the broader COVID-19 family project from which the current sample was drawn. The seven items were selected to capture core aspects of parent–child conflict (e.g., arguing, criticism, and general friction) while reducing participant burden during the lockdown period. The translation from English to Chinese followed standard forward- and back-translation procedures, and the content validity of the translated items was reviewed by experts in developmental psychology. Adolescents rated each item on a 5-point scale from 1 = not at all true to 5 = absolutely true (e.g., “My parents and I always seem to be arguing with each other”). The exact items used in the current study can be found in Appendix A. Mean scores were calculated, with higher scores indicating a higher level of parent–child conflict. Confirmatory factor analysis supported mixed evidence of model fit (CFI = 0.95, TLI = 0.93, RMSEA = 0.09, SRMR = 0.03), with the RMSEA indicating non-trivial misfit despite strong standardized loadings ranging from 0.79 to 0.87. The largest modification indices concerned potential residual covariances between Items 2 and 5, Items 2 and 3, and Items 5 and 6. This pattern suggests content-specific local dependence involving global or evaluative appraisals of the parent–child relationship and experiences specifically related to parental punishment, criticism, and anger. Internal reliability was α = 0.94, and McDonald’s Ω was 0.94 in the current sample. The mean inter-item correlation was 0.71, indicating substantial homogeneity and possible redundancy among the seven items.

#### 2.3.4. Covariates

The following variables were included as covariates because they had been associated with depressive symptoms during COVID-19: age, COVID-19-related events among relatives or close friends (0 = no, 1 = yes), COVID-19-related events in the community (0 = no, 1 = yes), and household SES. Parents reported their own and their spouses’ educational attainment (0 = middle school or below, 1 = high school or technical secondary school, 2 = bachelor’s degree, and 3 = master’s degree or above) and monthly income (0 = less than ¥2000, 1 = ¥2000 to less than ¥5000, 2 = ¥5000 to less than ¥10,000, 3 = ¥10,000 to less than ¥15,000, 4 = ¥15,000 to less than ¥20,000, and 5 = ¥20,000 or more). Maternal education, paternal education, maternal income, and paternal income were each standardized as z scores and then averaged to form the household SES composite; consequently, the composite did not itself have SD = 1. Higher scores indicate higher SES. The exact items are reported in Appendix A.4.

### 2.4. Statistical Analyses

Descriptive statistics, correlations, and gender and grade differences in the focal variables were examined in SPSS 23.0. Measurement and structural models were estimated in Mplus 8.3. Data were missing for age (n = 8, 0.31%), grade (n = 2, 0.08%), household SES (n = 612, 23.92%), and class identifiers (n = 8, 0.31%); no data were missing for the remaining variables. One adolescent was missing both age and class identifier, so the two counts corresponded to 15 unique excluded cases and an analytic sample of 2543. Missing data on endogenous variables were handled using full-information maximum likelihood. Because household SES was unavailable for some cases due to incomplete parent–child survey linkage, it was incorporated as a continuous endogenous variable through an auxiliary distribution equation. Under maximum likelihood, this specification assumes that the continuous endogenous variables follow a joint multivariate normal distribution and that household SES is missing at random conditional on the variables included in the model. Cases missing class identifiers or the fully exogenous age covariate could not be retained. All structural models were adjusted for age, household SES, COVID-19-related events in the community, and COVID-19-related events among relatives or close friends.

Before testing the structural model, gender measurement invariance was examined by successively testing configural, metric, and scalar models, with invariance evaluated using changes in CFI, RMSEA, and SRMR (Chen, 2007). A joint three-factor measurement model was then estimated, with preventive practices and parent–child conflict represented by their individual items and depressive symptoms represented by four parcels. Discriminant validity was evaluated using standardized factor loadings, latent factor correlations and their 95% confidence intervals, and average variance extracted (Fornell & Larcker, 1981). These latent factors were subsequently used in the structural analyses.

The class-level intraclass correlations (ICCs) were 0.012 for preventive practices, 0.023 for parent–child conflict, and 0.050 for depressive symptoms. The corresponding school-level ICCs were 0.000, 0.025, and 0.056, respectively. Because classes were the immediate sampling units and the sample included 80 classes but only four schools, class was selected as the clustering unit. Accordingly, all CFA, measurement invariance, and structural models used TYPE = COMPLEX RANDOM to obtain cluster-robust standard errors. The latent moderated mediation model was estimated using ML with numerical integration. Conditional indirect associations and their differences (the index of moderated mediation) were evaluated using 5000 cluster bootstrap replications, and percentile bootstrap confidence intervals were used as the primary inferential criterion (MacKinnon et al., 2004). Because no stratification variable was specified, the 80 classes were treated as belonging to a single stratum. Following the Mplus complex-sampling bootstrap procedure, each bootstrap replicate selected K − 1 = 79 classes with replacement from the original 80 classes and constructed replicate weights based on the number of times each class was selected. Mplus reported that all 5000 requested bootstrap replications were completed, and TECH9 indicated no estimation errors.

Because conventional outcome R2 estimates were unavailable for the latent interaction model, parallel observed-score regression models were estimated using the same analytic sample, covariates, and class-cluster adjustment. Models with and without the relevant gender interaction terms were compared to obtain sensitivity estimates of ΔR2.

Regression-based sensitivity analyses were conducted in G*Power 3.1 to evaluate the smallest incremental interaction effects detectable with 80% power and α = 0.05 (Faul et al., 2009). Four additional sensitivity analyses were conducted. First, to assess whether the treatment of missing household SES affected the findings, the primary moderated mediation model was re-estimated without household SES. Second, an alternative model specified depressive symptoms as the predictor, parent–child conflict as the mediator, and preventive practices as the outcome, using the same covariates, cluster adjustment, and 5000 bootstrap replications. Third, the moderated mediation model was estimated for primary- and middle-school students using a known-class multiple-group specification (Muthén & Muthén, 1998–2017). Factor loadings were constrained to equality across grade groups, whereas item intercepts were freely estimated; grade differences in the structural paths and conditional indirect associations were tested directly. Finally, to address the overlap with J. Liu et al. (2021), we re-estimated the latent moderated mediation model after excluding all 1594 adolescents included in that earlier study. Household SES was omitted because it was available for only 353 of these 958 adolescents (36.8%). The remaining covariates and class-level cluster adjustment were retained.

The covariance matrix of the main variables and the Mplus Syntax for the Primary Model can be found in the Supplementary Materials.

## 3. Results

### 3.1. Descriptive Analyses

The means and standard deviations of all study variables are presented in Table 1. An exploratory two-way multivariate analysis of variance (MANOVA) was conducted using the 2556 adolescents with complete data on gender, grade, and the three focal variables. Exploratory multivariate analysis of variance (MANOVA) revealed significant main effects of gender (0 = boys, 1 = girls) (Wilks’ λ = 0.99, F(3, 2550) = 9.83, *p* < 0.001, partial η^2^ = 0.011) and grade (0 = primary school, 1 = middle school) (Wilks’ λ = 0.96, F(3, 2550) = 39.63, *p* < 0.001, partial η^2^ = 0.045) and an interaction effect between gender and grade (Wilks’ λ = 0.996, F(3, 2550) = 3.05, *p* = 0.028, partial η^2^ = 0.004).

Follow-up univariate tests from the two-way MANOVA, which included gender, grade, and the gender × grade interaction, indicated that, compared with girls, boys reported significantly higher levels of parent–child conflict (F(1, 2552) = 4.81, *p* = 0.028, partial η^2^ = 0.002) and lower levels of preventive practices (F(1, 2552) = 15.13, *p* < 0.001, partial η^2^ = 0.006) during the COVID-19 outbreak period. Descriptively, parent–child conflict scores were *M* = 2.03 (*SD* = 1.01) for boys and *M* = 1.97 (*SD* = 0.99) for girls; preventive practice scores were *M* = 4.22 (*SD* = 0.70) for boys and *M* = 4.35 (*SD* = 0.64) for girls; and depressive symptom scores were *M* = 10.87 (*SD* = 9.63) for boys and *M* = 11.63 (*SD* = 10.01) for girls.

The univariate grade effects were significant for depressive symptoms (F(1, 2552) = 116.33, *p* < 0.001, partial η^2^ = 0.044) and parent–child conflict (F(1, 2552) = 47.36, *p* < 0.001, partial η^2^ = 0.018), with middle-school students reporting higher levels of both than primary-school students.

Moreover, as the central concept of the current study, preventive practice scores were negatively skewed among both boys (skewness = −0.57, kurtosis = −0.60) and girls (skewness = −0.88, kurtosis = 0.45). At the item level, the proportions selecting the maximum response ranged from 45.08% to 65.45% among boys and from 47.23% to 68.48% among girls. Overall, 29.14% of boys and 31.28% of girls scored the maximum on all three items. Thus, although girls showed slightly greater score restriction, the gender difference in the overall ceiling proportion was modest.

Bivariate correlations among all study variables are also presented in Table 1. Youth preventive practices were significantly and negatively associated with parent–child conflict and depressive symptoms. Parent–child conflicts were significantly and positively associated with depressive symptoms.

### 3.2. Gender Measurement Invariance

For preventive practices, metric invariance was supported (ΔCFI = 0.000, ΔRMSEA = 0.000, ΔSRMR = 0.023). Full scalar invariance was not met (ΔCFI = −0.004, ΔRMSEA = 0.026, ΔSRMR = 0.008), with modification indices indicating non-invariance in the first item intercept among girls. After freeing this intercept, the partial scalar model fit well (χ^2^(3) = 2.17, *p* = 0.538, CFI = 1.000, RMSEA = 0.000, SRMR = 0.024) and showed negligible change from the metric model (ΔCFI = 0.000, ΔRMSEA = 0.000, ΔSRMR = 0.001), supporting partial scalar invariance. In the partial scalar invariance model, the estimated latent variance of preventive practices was 0.335 among boys and 0.276 among girls, and the difference was not statistically significant (Wald χ^2^(1) = 2.53, *p* = 0.112).

Parent–child conflict demonstrated both metric (ΔCFI = −0.005, ΔRMSEA = −0.005, and ΔSRMR = 0.002) and scalar invariance (ΔCFI = −0.005, ΔRMSEA = −0.004, and ΔSRMR = 0.002). Depressive symptoms also demonstrated metric invariance (ΔCFI = −0.002, ΔRMSEA = −0.001, and ΔSRMR = 0.008) and scalar invariance (ΔCFI = −0.006, ΔRMSEA = 0.001, and ΔSRMR = −0.001). Thus, metric invariance was established for all three constructs, supporting comparisons of their structural associations across gender.

### 3.3. Measurement Model

The joint three-factor measurement model demonstrated acceptable fit (robust χ^2^(74) = 632.65, *p* < 0.001, CFI = 0.97, TLI = 0.96, RMSEA = 0.05, 90% CI [0.05, 0.06], and SRMR = 0.02). The standardized factor loadings ranged from 0.61 to 0.78 for preventive practices, from 0.79 to 0.87 for parent–child conflict, and from 0.85 to 0.90 for the depressive symptom parcels; all loadings were significant at *p* < 0.001. Moreover, the latent correlations were −0.27, 95% CI [−0.32, −0.21] between preventive practices and parent–child conflict; −0.36, 95% CI [−0.42, −0.31] between preventive practices and depressive symptoms; and 0.62, 95% CI [0.58, 0.65] between parent–child conflict and depressive symptoms. The corresponding AVEs were 0.51, 0.71, and 0.78. Each construct’s AVE exceeded its squared correlations with the other constructs, supporting their empirical separability.

### 3.4. Latent Moderated Mediation Analysis

Table 2 presents the unstandardized coefficients for the parent–child conflict and depressive symptom equations. The joint Wald test of the three gender interaction terms was significant (χ^2^(3) = 11.46, *p* = 0.010), indicating that at least one of the modeled associations differed by gender.

After the covariates were controlled, preventive practices were negatively associated with depressive symptoms (*B* = −0.20, *p* < 0.001). Adolescents’ preventive practices were negatively related to parent–child conflict (*B* = −0.50, *p* < 0.001). Youth parent–child conflict was positively associated with their depressive symptoms (*B* = 0.22, *p* < 0.001).

The preventive practices × gender interaction was not significant (*B* = 0.15, *p* = 0.088), indicating that this association did not reliably differ by gender. However, both interaction terms in the depressive symptom equation were significant: preventive practices × gender (*B* = 0.08, *p* = 0.037) and parent–child conflict × gender (*B* = 0.07, *p* = 0.002).

Because conventional R^2^ estimates were unavailable for the latent interaction model, parallel observed-score sensitivity models were estimated. Adding the interaction terms increased the explained variance in parent–child conflict from 0.074 to 0.077 (ΔR^2^ = 0.003) and that in depressive symptoms from 0.376 to 0.380 (ΔR^2^ = 0.004).

Probing the interactions showed that the negative association between preventive practices and depressive symptoms was stronger for boys (*B* = −0.20, *p* < 0.001) than for girls (*B* = −0.13, *p* < 0.001), whereas the positive association between parent–child conflict and depressive symptoms was stronger for girls (*B* = 0.29, *p* < 0.001) than for boys (*B* = 0.22, *p* < 0.001). The conditional indirect association through parent–child conflict was significant for both boys (*B* = −0.11, *p* < 0.001, 95% CI [−0.14, −0.08]) and girls (*B* = −0.10, *p* < 0.001, 95% CI [−0.14, −0.06]). However, the difference between these indirect associations was not significant (index of moderated mediation = 0.01, *p* = 0.766). The conditional total associations were significant for boys (*B* = −0.31, *p* < 0.001) and girls (*B* = −0.23, *p* < 0.001), but their difference did not reach significance (Δ*B* = 0.08, *p* = 0.054, 95% CI [−0.003, 0.166]). Taken together, the indirect association represented 34.6% of the total association among boys and 44.5% among girls.

Regression-based sensitivity analyses indicated that, with an analytic sample of 2543, α = 0.05, and 80% power, the minimum detectable incremental effect was *f*^2^ = 0.003 for the interaction predicting parent–child conflict and *f*^2^ = 0.004 for the two interaction terms jointly predicting depressive symptoms. Thus, the study was sufficiently powered to detect very small interaction effects, and statistically significant interactions should be interpreted in terms of their modest magnitude.

### 3.5. Sensitivity Analyses

Omitting household SES produced virtually unchanged interaction estimates. The preventive practices × gender association with parent–child conflict was *B* = 0.145, the preventive practices × gender association with depressive symptoms was *B* = 0.078, and the parent–child conflict × gender association with depressive symptoms was *B* = 0.071, compared with 0.147, 0.077, and 0.071, respectively, in the primary model. The difference between the conditional indirect associations also remained nonsignificant.

The alternative ordering of depressive symptoms → parent–child conflict → preventive practices was additionally examined. Depressive symptoms were positively associated with parent–child conflict (*B* = 1.33, 95% CI [1.21, 1.45]). However, after accounting for depressive symptoms, parent–child conflict was not significantly associated with preventive practices (*B* = −0.04, *p* = 0.061, 95% CI [−0.08, 0.002]). Accordingly, the reverse indirect association was not significant (*B* = −0.050, *p* = 0.057, 95% CI [−0.100, 0.003]).

The grade sensitivity analysis included students from two primary schools (*n* = 935) and two middle schools (*n* = 1608). The indirect association was significant for boys and girls in both grade groups but did not differ significantly between primary and middle school for either boys (Δ*B* = −0.02, *p* = 0.53) or girls (Δ*B* = −0.02, *p* = 0.55). The gender difference in the indirect association also did not vary by grade (Δ*B* = −0.004, *p* = 0.93). Because the grade groups corresponded to two primary and two middle schools, this analysis also provided a partial check on school composition. Overall, the central indirect pattern was consistent across grade levels.

We next re-estimated the model after excluding all 1594 adolescents who had been included in the study of J. Liu et al. (2021). Of the 964 nonoverlapping adolescents, 958 had the required class identifier and fully exogenous covariate data and were retained in the analysis. The central pattern was reproduced: preventive practices were negatively associated with parent–child conflict and depressive symptoms, whereas parent–child conflict was positively associated with depressive symptoms, among both boys and girls. The conditional indirect association was significant among boys (*B* = −0.18) and girls (*B* = −0.27). Their difference remained nonsignificant (*B* = −0.09). However, the preventive practices × gender interaction predicting depressive symptoms and the parent–child conflict × gender interaction predicting depressive symptoms were no longer statistically significant. Thus, the nonoverlapping-sample analysis reproduced the central direct and indirect associations but did not independently support the path-specific gender moderation found in the primary analysis.

## 4. Discussion

The current study focused on the concurrent association between preventive practices and depressive symptoms among Chinese youth during the initial COVID-19 lockdown. We examined a moderated mediation model linking preventive practices, parent–child conflict, and depressive symptoms (see Figure 1). Preventive practices were negatively associated with depressive symptoms, and a significant indirect association was observed through parent–child conflict. In the full-sample analysis, the association between preventive practices and depressive symptoms was more negative for boys, whereas the association between parent–child conflict and depressive symptoms was stronger for girls. However, these path-specific gender differences were not statistically significant in the nonoverlapping-subsample analysis.

### 4.1. Preventive Practices, Parent–Child Conflict, and Depressive Symptoms

Results from the covariate-adjusted latent structural model indicated that adolescents’ preventive practices were negatively associated with their depressive symptoms, supporting Hypothesis 1. The outbreak of COVID-19 was likely experienced as a stressor that was largely outside adolescents’ control (Sitanggang et al., 2021). From a stress and coping perspective, preventive practices may have provided concrete actions through which to respond to uncertainty, potentially strengthening perceived control (Lazarus & Folkman, 1984; Zhang et al., 2020). This interpretation is consistent with previous findings among adults (C. Wang et al., 2020). However, because coping appraisals and perceived control were not assessed, they are possible explanations for the observed association rather than mechanisms demonstrated by the present findings.

The significant indirect associations through parent–child conflict were also consistent with the proposed family process account. As the primary socialization agent, parents had the responsibility of teaching their children how to protect themselves during the pandemic (Peplak et al., 2021). Prior research suggests that, when adolescents do not follow preventive guidance, parents may experience greater concern and respond with more controlling behavior to promote compliance (Cavicchiolo et al., 2021), potentially contributing to parent–child conflict. Indeed, Bülow et al. (2021) found that, compared with parental self-report, adolescents reported higher levels of perceived psychologically and behaviorally controlling parental practice during the COVID-19 lockdown period. Conversely, adolescents’ engagement in preventive practices may reflect greater alignment with parent-endorsed health rules and may therefore be associated with less opposition and conflict. Similarly, parental enforcement and control, and parents’ awareness of adolescents’ preventive behavior were not assessed. This account should therefore be regarded as a possible family process explanation rather than a mechanism demonstrated by the present data.

It should be noted that the current cross-sectional design also permits alternative interpretations of this association. For instance, adolescents experiencing more depressive symptoms may have had less motivation to implement preventive practices, while adolescents in more conflictual family relationships may have been more likely to resist parent-endorsed health rules. Recent longitudinal and daily report family studies suggest that family functioning may precede health-protective adherence and that implementing health preventive practices can coincide with both relational strain and closeness (Bai et al., 2024; Fosco et al., 2023). However, in the alternative model examined in the present study, the reverse indirect association from depressive symptoms to preventive practices through conflict was not significant. This nonsignificant reverse indirect association should not be interpreted as validating the proposed ordering. Because all constructs were assessed concurrently, the data cannot distinguish the proposed sequence from this reverse sequence or other plausible temporal orderings (Maxwell & Cole, 2007; Maxwell et al., 2011). Parent–child conflict should therefore be understood as a concurrent family process correlate linking preventive practices with depressive symptoms rather than as an established mechanism.

### 4.2. Gender Effects

Consistent with previous studies (Xue et al., 2021), boys reported less engagement in preventive practices than girls during the COVID-19 outbreak. In the full-sample analysis, gender did not significantly moderate the association between preventive practices and parent–child conflict. However, the negative direct association between preventive practices and depressive symptoms was stronger for boys, whereas the positive association between parent–child conflict and depressive symptoms was stronger for girls. The point estimates followed the same directions in the nonoverlapping subsample, but neither of the latter two interaction effects remained statistically significant. This pattern suggests that the path-specific gender differences may be more sensitive to sample composition and family context. More specifically, because inclusion in the study of J. Liu et al. (2021) required linked maternal data, excluding those participants produced a differently constituted sample comprising adolescents with father-completed parent questionnaires and adolescents without linked parent questionnaires. These groups reflect different patterns of caregiver participation in the study and may encompass different caregiving arrangements and parent–adolescent interaction contexts during the lockdown.

Moreover, the observed-score sensitivity models indicated that the interaction terms accounted for only an additional 0.3% of the variance in parent–child conflict and 0.4% of the variance in depressive symptoms. In addition, girls’ preventive practice scores showed slightly less observed variability than boys’ scores (SD = 0.64 vs. 0.70). The corresponding latent variance was also numerically smaller among girls than among boys (0.276 vs. 0.335), but this difference was not statistically significant (Wald χ^2^(1) = 2.53, *p* = 0.112), suggesting limited evidence of pronounced differential range restriction. Nevertheless, because latent modeling corrects attenuation due to unreliability but not restriction in true score variance, differential range restriction cannot be completely ruled out. The interaction should consequently be interpreted as small and tentative, rather than as definitive evidence of a stronger association for boys.

The mean-level gender difference in preventive practices may be considered in light of gender socialization in the Chinese context. Chinese parents have been described as fostering greater dependency and tenderness in girls while expecting greater independence and autonomy from boys (Shek, 2002). Moreover, Chinese adolescent girls have reported greater parental demandingness than boys (Shek, 2008). These perspectives may help contextualize girls’ higher mean engagement in preventive practices. By contrast, the stronger positive association between parent–child conflict and depressive symptoms among girls is consistent with findings obtained before and during the COVID-19 pandemic (Lewis et al., 2014; J. Liu et al., 2021). Gender role perspectives suggest that girls may place greater emphasis on interpersonal intimacy and be more emotionally affected by relational disharmony (Hill & Lynch, 1983; Sigfusdottir et al., 2004). It should be noted that this interpretation remains tentative, as the relevant socialization and relational sensitivity constructs were not measured in the current study.

### 4.3. Limitations and Future Research Directions

Several limitations of the current study should be noted. First, the data were collected from youth in Henan, China, during the initial, highly restrictive phase of the pandemic. Nations and regions differed in outbreak severity and in the acceptance and interpretation of preventive practices (Wen et al., 2022), so the applicability of the model elsewhere requires examination. Online recruitment during school closure also meant that the invitation denominator and information about nonparticipants were unavailable, preventing direct evaluation of participation and self-selection. In addition, class-level clustering did not separately model the residual school-level dependence in depressive symptoms (school ICC = 0.056). With only four schools, reliable school-level random-effects inference was not feasible. The interaction *p* values of 0.037 and 0.002 should therefore be interpreted cautiously. The grade group sensitivity analysis partly addressed school composition because the primary- and middle-school groups each comprised two schools, but it was not a substitute for school fixed-effects analysis. The two path-specific gender interactions identified in the full-sample analysis were also not statistically significant in the nonoverlapping subsample. Accordingly, these gender-specific findings may depend partly on sample composition and require replication across different family and caregiver contexts.

Second, all focal constructs were reported by adolescents on the same occasion. Although the joint three-factor measurement model supported their empirical separability, this evidence does not eliminate construct overlap or common-method bias (Podsakoff et al., 2003). The seven-item PEQ model showed non-trivial local misfit (RMSEA = 0.09) despite high loadings. The content provides a plausible substantive explanation: some items describe disagreement, arguing, punishment, and resistance, whereas others are global negative appraisals of relationship quality. The mean inter-item correlation of 0.71 also suggests substantial homogeneity and possible redundancy. Accordingly, the parent–child conflict score combines conflict occurrence with negative relationship appraisal, and its association with depressive symptoms may be inflated by shared negative affect, appraisal, and method variance. The parent questionnaire included demographic information but no independent measure of parent–child conflict or adolescent adjustment. Future research should incorporate parent reports, observed parent–adolescent interactions, and repeated assessments of specific conflict events.

Third, the three-item preventive practices factor had modest internal consistency. It should therefore be interpreted as a narrow indicator of reported engagement or compliance, not as a validated measure of autonomous coping or general health behavior. Finally, the current model did not include several plausible correlates of depressive symptoms, including pre-existing symptoms, loneliness, peer and school disruption, parental mental health, and broader pandemic stress. Parent–child conflict should therefore be understood as one correlate within a multifactorial context of adolescents’ depressive symptoms.

### 4.4. Implications

The present study extends research on the psychological and family correlates of adolescents’ preventive practices during the initial COVID-19 outbreak in China. Greater engagement in preventive practices co-occurred with less parent–child conflict and fewer depressive symptoms, although the cross-sectional design does not establish that adherence reduced either conflict or depressive symptoms. These findings are not meant to suggest that the specific COVID-19 preventive behaviors studied are still necessary today. Instead, they should be understood as reflecting a more general situation in which adolescents are asked to follow new health-related rules or instructions that are supported and encouraged by their parents. Because adolescence is characterized by increasing autonomy, the ways in which such instructions are explained, accepted, and negotiated may be closely related to parent–child conflict. Self-determination and social-domain perspectives suggest that providing clear rationales, acknowledging adolescents’ perspectives, and respecting their developing autonomy may facilitate the acceptance and internalization of health guidance (Ntoumanis et al., 2021; Smetana & Rote, 2019; Soenens et al., 2007). Future public health guidance and family-based initiatives may therefore benefit from considering how health directives are communicated and negotiated with adolescents, while longitudinal and intervention research is needed to determine whether these communication processes improve adherence, family relationships, or adolescent mental health.

## 5. Conclusions

Among Chinese adolescents surveyed during the initial COVID-19 outbreak, greater engagement in preventive practices was associated with fewer depressive symptoms, with lower parent–child conflict statistically accounting for part of this association. The indirect association through parent–child conflict was significant for both boys and girls, but its magnitude did not differ significantly by gender. Beyond the specific practices examined during COVID-19, the findings suggest that adolescents’ behavioral responses and family relationships should be considered together when families negotiate unfamiliar health-related requirements.

## Figures and Tables

**Table 1:** Descriptive statistics and bivariate correlations among study variables.

Variable	Valid N	M (SD)	Range	1	2	3	4	5	6	7
1. Gender (1 = girls)	2558	-	0, 1							
2. COVID-19-related events among relatives/close friends (1 = yes)	2558	-	0, 1	0.006						
3. COVID-19-related events in the community (1 = yes)	2558	-	0, 1	0.041 *	0.176 **					
4. Age	2550	13.06 (1.56)	10–16	−0.015	−0.004	0.002				
5. Household SES	1946	0.00 (0.73)	−1.16–2.76	0.029	0.019	0.043	−0.213 **			
6. Preventive practices	2558	4.29 (0.67)	1–5	0.087 **	−0.031	−0.010	−0.044 *	0.072 **		
7. Parent–child conflict	2558	2.00 (1.00)	1–5	−0.032	0.028	0.094 **	0.104 **	−0.006	−0.236**	
8. Depressive symptoms	2558	11.24 (9.82)	0–60	0.039	0.067 **	0.166 **	0.150 **	−0.057 *	−0.279 **	0.573 **

Note: Correlations are zero-order associations estimated using pairwise-complete observations; pairwise sample sizes varied because of missing age and household SES data. For binary variables, the table reports valid N and the coding range; continuous variables are presented as M (SD). Household SES has SD = 0.73 rather than 1.00 because it is the average of four standardized components. M = mean; SD = standard deviation; SES = socioeconomic status. Missing household SES data primarily reflected unavailable parent-completed questionnaires. * *p* < 0.05; ** *p* < 0.01.

**Table 2:** Unstandardized coefficients and conditional indirect associations from the latent moderated mediation model.

**Predictor/Group**	***B***	**SE**	**z**	**95% Percentile Bootstrap CI**
Outcome: Parent–child conflict				
Age	0.07	0.01	6.90	[0.04, 0.08]
Household SES	0.04	0.04	1.12	[−0.03, 0.10]
COVID-19-related events in the community	0.27	0.07	3.73	[0.13, 0.41]
COVID-19-related events among relatives or close friends	0.02	0.20	0.11	[−0.37, 0.39]
Preventive practices	−0.50	0.07	−7.33	[−0.64, −0.37]
Gender	−0.04	0.04	−1.03	[−0.11, 0.04]
Preventive practices × gender	0.15	0.09	1.71	[−0.02, 0.32]
Outcome: Depressive symptoms				
Age	0.02	0.01	4.30	[0.01, 0.03]
Household SES	−0.02	0.01	−1.69	[−0.04, 0.004]
COVID-19-related events in the community	0.15	0.03	4.99	[0.09, 0.20]
COVID-19-related events among relatives or close friends	0.13	0.08	1.54	[−0.03, 0.29]
Preventive practices	−0.20	0.03	−7.80	[−0.26, −0.15]
Parent–child conflict	0.22	0.02	13.29	[0.18, 0.25]
Gender	−0.01	0.02	−0.39	[−0.05, 0.04]
Preventive practices × gender	0.08	0.04	2.09	[0.01, 0.15]
Parent–child conflict × gender	0.07	0.02	3.16	[0.03, 0.11]
Conditional indirect associations				
**Group**	**Indirect association**	**SE**	**z**	**95% percentile bootstrap CI**
Boys	−0.11	0.02	−6.57	[−0.14, −0.08]
Girls	−0.10	0.02	−4.95	[−0.14, −0.06]
Difference (girls−boys)	0.01	0.02	0.30	[−0.04, 0.05]

Note: *B* = unstandardized coefficient; SE = cluster bootstrap standard error; z = estimate/SE; CI = 95% percentile cluster bootstrap confidence interval based on 5000 requested replications. Latent factors were scaled by fixing the loading of the first listed indicator to 1. Gender was coded as 0 = boys and 1 = girls; therefore, interaction coefficients represent girls minus boys. Covariates included age, COVID-19-related events among relatives or close friends, COVID-19-related events in the community, and household SES. Because only 1.05% of adolescents reported COVID-19-related events among relatives or close friends, this covariate provided limited information and was retained as an adjustment variable.

## Data Availability

Individual-level data cannot be publicly shared because the participants were minors and the informed consent did not authorize public data release. The Mplus analysis syntax and aggregate covariance matrix are provided in Supplementary Material S1.

## Note

1 In the item-level CFA and measurement-invariance models for depressive symptoms, residual covariances among the four positively worded, reverse-scored CES-DC items (Items 4, 8, 12, and 16) were freely estimated to account for shared wording-related method variance.

## Supplementary Materials

The following supporting information can be downloaded at: https://www.mdpi.com/article/10.3390/bs16091654/s1, Table S1. Variables and estimated descriptive statistics; Table S2. Covariances involving Variables 1–10; Mplus Syntax for the Primary Model.

## Appendix A

### Appendix A.1. Depressive Symptoms

Measure: Chinese version of the Center for Epidemiologic Studies Depression Scale (CES-D; Radloff, 1977).
Response scale: Items were rated on a 4-point Likert scale ranging from 0 (not at all) to 3 (a lot).
The English wording below provides item-by-item translations of the Chinese statements that were administered. These translations are presented to permit cross-language verification and are not intended to reproduce the original English CES-D items verbatim.

**Items in English**	**Items in Chinese**
1. I was bothered by things that usually do not bother me.	一些通常并不困扰我的事使我心烦
2. I did not feel like eating; my appetite was poor.	我不想吃东西, 胃口不好
3. I felt that I could not shake off my distress even with help from my family and friends.	我觉得即使有家人和朋友帮助也无法摆脱苦闷
4. I felt that I was just as good as other people.*	我感觉和别人一样好*
5. I had difficulty concentrating on what I was doing.	我很难集中精力做事情
6. I felt depressed.	我感到压抑
7. Everything I did felt effortful.	我感到做什么事都很吃力
8. I felt hopeful about the future.*	我对未来充满希望*
9. I felt that my life was worthless.	我认为我的生活一无是处
10. I felt fearful.	我感到恐惧
11. My sleep was restless.	我睡不安稳
12. I was happy.*	我很开心*
13. I talked less than usual.	我比平时话更少
14. I felt lonely.	我感到孤独
15. People were unfriendly to me.	人们对我不友好
16. I felt happy with my life..*	我生活幸福 *
17. I cried loudly.	我大声哭
18. I felt sad.	我感到悲伤
19. I felt that people disliked me.	我觉得别人不喜欢我
20. I walked slowly.	我走路很慢
*Note:* * reverse-scored item.	

### Appendix A.2. Preventive Practices

Response scale: Items were rated on a 5-point Likert scale ranging from 1 (not at all) to 5 (very much).

**Items in English**	**Items in Chinese**
1. I paid attention to disease prevention guidelines.	1. 我主动关注新冠疫情的事件发展和防治消息
2. I wore a facial mask or took other necessary preventive actions when going out.	2. 我出门时会带上口罩或进行其他防护措施
3. I washed my hands more often.	3. 我比平时更频繁地洗手和注意卫生

### Appendix A.3. Parent–Child Conflict

Measure: Conflict subscale of the Parental Environment Questionnaire (PEQ; Elkins et al., 1997).
Response scale: Items were rated on a 5-point Likert scale ranging from 1 (not at all true) to 5 (absolutely true).

**Items in English**	**Items in Chinese**
1. My parents and I always seem to struggle with each other.	1. 父母和我似乎总是在相互斗争
2. I have a bad relationship with my parents.	2. 我和父母关系很糟糕
3. I feel that my parents treat me unfairly.	3. 我觉得父母对我不公平
4. My parents and I often disagree and sometimes argue.	4. 我和父母经常看法不一致，有时还会争吵
5. I feel angry about my parents’ punishments and sometimes resist them.	5. 我对父母的处罚感到愤怒，有时会反抗
6. I feel that my parents are always punishing and criticizing me.	6. 我觉得父母总是在惩罚和批评我
7. I find it very difficult to get along with my parents.	7. 与父母相处使我感到很费劲

### Appendix A.4. Covariates

Age: Age in years.
COVID-19-related events among relatives or close friends: Did any relatives or close friends become infected with COVID-19, undergo quarantine, or die during the COVID-19 outbreak? Response options: No; Yes.
COVID-19-related events in the community: Did anyone in your community become infected with COVID-19, undergo quarantine, or die during the COVID-19 outbreak? Response options: No; Yes.
Parental education (parent-reported): Your educational level/your spouse’s educational level: middle school or below; high school or technical secondary school; bachelor’s degree; master’s degree or above.
Household income (parent-reported): Your monthly income/your spouse’s monthly income: less than ¥2000; ¥2000 to less than ¥5000; ¥5000 to less than ¥10,000; ¥10,000 to less than ¥15,000; ¥15,000 to less than ¥20,000; ¥20,000 or more.

## References

[B1-behavsci-16-01654] Aydin F., Kaya F. (2021). Does compliance with the preventive health behaviours against COVID-19 mitigate the effects of depression, anxiety and stress?. Studies in Psychology.

[B2-behavsci-16-01654] Bai S., Tornello S. L., Mogle J. A., Feinberg M. E. (2024). Daily implementation of health-protective behaviors and family life during the early months of the COVID-19 pandemic. Family Process.

[B3-behavsci-16-01654] Bandura A. (1977). Self-efficacy: Toward a unifying theory of behavioral change. Psychological Review.

[B4-behavsci-16-01654] Branje S., Morris A. S. (2021). The impact of the COVID-19 pandemic on adolescent emotional, social, and academic adjustment. Journal of Research on Adolescence.

[B5-behavsci-16-01654] Burak D. (2023). The effect of risk and protective factors on primary school students’ COVID-19 anxiety: Back to school after the pandemic. Child Indicators Research.

[B6-behavsci-16-01654] Bülow A., Keijsers L., Boele S., van Roekel E., Denissen J. J. A. (2021). Parenting adolescents in times of a pandemic: Changes in relationship quality, autonomy support, and parental control?. Developmental Psychology.

[B7-behavsci-16-01654] Cavicchiolo E., Manganelli S., Girelli L., Cozzolino M., Lucidi F., Alivernini F. (2021). Adolescents at a distance: The importance of socio-cognitive factors in preventive behavior during the COVID-19 pandemic. European Journal of Health Psychology.

[B8-behavsci-16-01654] Chen F. F. (2007). Sensitivity of goodness of fit indexes to lack of measurement invariance. Structural Equation Modeling: A Multidisciplinary Journal.

[B9-behavsci-16-01654] Cheng C., Cheung M. W. L. (2005). Psychological responses to outbreak of severe acute respiratory syndrome: A prospective, multiple time-point study. Journal of Personality.

[B10-behavsci-16-01654] Cvetković V. M., Nikolić N., Radovanović Nenadić U., Öcal A., Noji E. K., Zečević M. (2020). Preparedness and preventive behaviors for a pandemic disaster caused by COVID-19 in Serbia. International Journal of Environmental Research and Public Health.

[B11-behavsci-16-01654] Elkins I. J., McGue M., Iacono W. G. (1997). Genetic and environmental influences on parent–son relationships: Evidence for increasing genetic influence during adolescence. Developmental Psychology.

[B12-behavsci-16-01654] Eysenbach G. (2004). Improving the quality of web surveys: The checklist for reporting results of internet E-surveys (CHERRIES). Journal of Medical Internet Research.

[B13-behavsci-16-01654] Faul F., Erdfelder E., Buchner A., Lang A.-G. (2009). Statistical power analyses using G*Power 3.1: Tests for correlation and regression analyses. Behavior Research Methods.

[B14-behavsci-16-01654] Fornell C., Larcker D. F. (1981). Evaluating structural equation models with unobservable variables and measurement error. Journal of Marketing Research.

[B15-behavsci-16-01654] Fosco G. M., Lee H., Feinberg M. E., Fang S., Sloan C. J. (2023). COVID-19 family dynamics and health protective behavior adherence: A 16-wave longitudinal study. Health Psychology.

[B16-behavsci-16-01654] Franko D. L., Thompson D., Bauserman R., Affenito S. G., Striegel-Moore R. H. (2008). What’s love got to do with it? Family cohesion and healthy eating behaviors in adolescent girls. International Journal of Eating Disorders.

[B17-behavsci-16-01654] Hill J. P., Lynch M. E., Brooks-Gunn J., Petersen A. C. (1983). The intensification of gender-related role expectations during early adolescence. Girls at puberty: Biological and psychosocial perspectives.

[B18-behavsci-16-01654] Jeon S., Lee D., Weems C. F. (2022). COVID-19 and family distancing efforts: Contextual demographic and family conflict correlates. Journal of Family Issues.

[B19-behavsci-16-01654] Lazarus R. S., Folkman S. (1984). Stress, appraisal, and coping.

[B20-behavsci-16-01654] Lewis G., Collishaw S., Thapar A., Harold G. T. (2014). Parent–child hostility and child and adolescent depression symptoms: The direction of effects, role of genetic factors, and gender. European Child & Adolescent Psychiatry.

[B21-behavsci-16-01654] Li F., Liang W., Rhodes R. E., Duan Y., Wang X., Shang B., Yang Y., Jiao J., Yang M., Supriya R., Baker J. S., Yi L. (2022). A systematic review and meta-analysis on the preventive behaviors in response to the COVID-19 pandemic among children and adolescents. BMC Public Health.

[B22-behavsci-16-01654] Liu C. H., Smiley P. A., Vicman J. M., Wong G. T. F., Doan S. N. (2022). The roles of life stress and preventive health behaviors on parent mental health during the COVID-19 pandemic. Journal of Health Psychology.

[B23-behavsci-16-01654] Liu J., Zhou T., Yuan M., Ren H., Bian X., Coplan R. J. (2021). Daily routines, parent–child conflict, and psychological maladjustment among Chinese children and adolescents during the COVID-19 pandemic. Journal of Family Psychology.

[B24-behavsci-16-01654] MacKinnon D. P., Lockwood C. M., Williams J. (2004). Confidence limits for the indirect effect: Distribution of the product and resampling methods. Multivariate Behavioral Research.

[B25-behavsci-16-01654] Madigan S., Racine N., Vaillancourt T., Korczak D. J., Hewitt J. M. A., Pador P., Park J. L., McArthur B. A., Holy C., Neville R. D. (2023). Changes in depression and anxiety among children and adolescents from before to during the COVID-19 pandemic: A systematic review and meta-analysis. JAMA Pediatrics.

[B26-behavsci-16-01654] Maxwell S. E., Cole D. A. (2007). Bias in cross-sectional analyses of longitudinal mediation. Psychological Methods.

[B27-behavsci-16-01654] Maxwell S. E., Cole D. A., Mitchell M. A. (2011). Bias in cross-sectional analyses of longitudinal mediation: Partial and complete mediation under an autoregressive model. Multivariate Behavioral Research.

[B28-behavsci-16-01654] Muthén L. K., Muthén B. O. (1998–2017). Mplus user’s guide.

[B29-behavsci-16-01654] Ntoumanis N., Ng J. Y. Y., Prestwich A., Quested E., Hancox J. E., Thøgersen-Ntoumani C., Deci E. L., Ryan R. M., Lonsdale C., Williams G. C. (2021). A meta-analysis of self-determination theory-informed intervention studies in the health domain: Effects on motivation, health behavior, physical, and psychological health. Health Psychology Review.

[B30-behavsci-16-01654] Park S., Oh S. (2022). Factors associated with preventive behaviors for COVID-19 among adolescents in South Korea. Journal of Pediatric Nursing.

[B31-behavsci-16-01654] Peplak J., Klemfuss J. Z., Yates T. M. (2021). Parent–adolescent conversations about COVID-19 influence adolescents’ empathic concern and adherence to health protective behaviors. Journal of Adolescent Health.

[B32-behavsci-16-01654] Podsakoff P. M., MacKenzie S. B., Lee J.-Y., Podsakoff N. P. (2003). Common method biases in behavioral research: A critical review of the literature and recommended remedies. Journal of Applied Psychology.

[B33-behavsci-16-01654] Qu Y., Li X., Ni B., He X., Zhang K., Wu G. (2021). Identifying the role of parent–child conflict and intimacy in Chinese adolescents’ psychological distress during school reopening in the COVID-19 pandemic. Developmental Psychology.

[B34-behavsci-16-01654] Radloff L. S. (1977). The CES-D scale: A self-report depression scale for research in the general population. Applied Psychological Measurement.

[B35-behavsci-16-01654] Ren H., Cheah C. S. L., Liu J. (2021). The cost and benefit of fear induction parenting on children’s health during the COVID-19 outbreak. Developmental Psychology.

[B36-behavsci-16-01654] Riiser K., Helseth S., Haraldstad K., Torbjørnsen A., Richardsen K. R. (2020). Adolescents’ health literacy, health protective measures, and health-related quality of life during the COVID-19 pandemic. PLoS ONE.

[B37-behavsci-16-01654] Rogers A. A., Ha T., Ockey S. (2021). Adolescents’ perceived socio-emotional impact of COVID-19 and implications for mental health: Results from a U.S.-based mixed-methods study. Journal of Adolescent Health.

[B38-behavsci-16-01654] Saurabh K., Ranjan S. (2020). Compliance and psychological impact of quarantine in children and adolescents due to COVID-19 pandemic. Indian Journal of Pediatrics.

[B39-behavsci-16-01654] Scholz U., Freund A. M. (2021). Determinants of protective behaviours during a nationwide lockdown in the wake of the COVID-19 pandemic. British Journal of Health Psychology.

[B40-behavsci-16-01654] Sharma V., Ortiz M. R., Sharma N. (2020). Risk and protective factors for adolescent and young adult mental health within the context of COVID-19: A perspective from Nepal. Journal of Adolescent Health.

[B41-behavsci-16-01654] Shek D. T. L. (2002). Chinese adolescents’ perceptions of family functioning: Personal, school-related and family correlates. Genetic, Social, and General Psychology Monographs.

[B42-behavsci-16-01654] Shek D. T. L. (2008). Perceived parental control and parent–child relational qualities in early adolescents in Hong Kong: Parent gender, child gender and grade differences. Sex Roles.

[B43-behavsci-16-01654] Sigfusdottir I. D., Farkas G., Silver E. (2004). The role of depressed mood and anger in the relationship between family conflict and delinquent behavior. Journal of Youth and Adolescence.

[B44-behavsci-16-01654] Sitanggang F. P., Wirawan G., Wirawan I., Lesmana C., Januraga P. P. (2021). Determinants of mental health and practice behaviors of general practitioners during the COVID-19 pandemic in Bali, Indonesia: A cross-sectional study. Risk Management and Healthcare Policy.

[B45-behavsci-16-01654] Smetana J. G., Asquith P. (1994). Adolescents’ and parents’ conceptions of parental authority and personal autonomy. Child Development.

[B46-behavsci-16-01654] Smetana J. G., Rote W. M. (2019). Adolescent–parent relationships: Progress, processes, and prospects. Annual Review of Developmental Psychology.

[B47-behavsci-16-01654] Soenens B., Vansteenkiste M., Lens W., Luyckx K., Goossens L., Beyers W., Ryan R. M. (2007). Conceptualizing parental autonomy support: Adolescent perceptions of promotion of independence versus promotion of volitional functioning. Developmental Psychology.

[B48-behavsci-16-01654] Tambling R. R., Tomkunas A. J., Russell B. S., Horton A. L., Hutchison M. (2021). Thematic analysis of parent–child conversations about COVID-19: “Playing it safe”. Journal of Child and Family Studies.

[B49-behavsci-16-01654] UNICEF China (2020). Student in Beijing resumes study in her living room.

[B50-behavsci-16-01654] Wang C., Pan R., Wan X., Tan Y., Xu L., Ho C. S., Ho R. C. (2020). Immediate psychological responses and associated factors during the initial stage of the 2019 coronavirus disease (COVID-19) epidemic among the general population in China. International Journal of Environmental Research and Public Health.

[B51-behavsci-16-01654] Wang L., Chen L., Jia F., Shi X., Zhang Y., Li F., Hao Y., Hou Y., Deng H., Zhang J., Huang L., Xie X., Fang S., Xu Q., Xu L., Guan H., Wang W., Shen J., Li F., Wang X. (2021). Risk factors and prediction nomogram model for psychosocial and behavioural problems among children and adolescents during the COVID-19 pandemic: A national multicentre study. Journal of Affective Disorders.

[B52-behavsci-16-01654] Wang M., Armour C., Wu Y., Ren F., Zhu X., Yao S. (2013). Factor structure of the CES-D and measurement invariance across gender in Mainland Chinese adolescents. Journal of Clinical Psychology.

[B53-behavsci-16-01654] Wang Y., Tang W. (2025). The association between parent–adolescent conflicts and depressive mood: A systematic review and meta-analysis. BMC Psychology.

[B54-behavsci-16-01654] Wen F., Ye H., Zuo B., Han S., Zhu J., Ke W., He Y. (2022). The association between insecurity and subjective well-being among youth during the COVID-19 outbreak: A moderated mediation model. Journal of Affective Disorders.

[B55-behavsci-16-01654] Wolf K., Schmitz J. (2024). Scoping review: Longitudinal effects of the COVID-19 pandemic on child and adolescent mental health. European Child & Adolescent Psychiatry.

[B56-behavsci-16-01654] Xue Q., Xie X., Liu Q., Zhou Y., Zhu K., Wu H., Wan Z., Feng Y., Meng H., Zhang J., Zuo P., Song R. (2021). Knowledge, attitudes, and practices towards COVID-19 among primary school students in Hubei province, China. Children and Youth Services Review.

[B57-behavsci-16-01654] Yuan J., Cao B., Zhang C., Chan P. S. F., Xin M., Fang Y., Chen Y., Huang D., Li L., Xuan X., Zhang G., Pan Y., He Y., Wang Z. (2022). Changes in compliance with personal preventive measures and mental health status among Chinese factory workers during the COVID-19 pandemic: An observational prospective cohort study. Frontiers in Public Health.

[B58-behavsci-16-01654] Zhang C., Ye M., Fu Y., Yang M., Luo F., Yuan J., Tao Q. (2020). The psychological impact of the COVID-19 pandemic on teenagers in China. Journal of Adolescent Health.

[B59-behavsci-16-01654] Zhengzhou Municipal Bureau of Statistics (2021a). 2020 statistical bulletin on the national economic and social development of Zhengzhou.

[B60-behavsci-16-01654] Zhengzhou Municipal Bureau of Statistics (2021b). Bulletin of the Seventh National Population Census of Zhengzhou (No. 4): Educational attainment of permanent residents.

