# Supplementary material for: COVID-19 Preventive Practices, Parent–Child Conflict, and Depressive Symptoms Among Chinese Adolescents
Source: Behav Sci (Basel). 2026 Sep 15;16(9):1654. doi: 10.3390/bs16091654 (PMC13603774; doi:10.3390/bs16091654)
Supplement: Supplementary file 1 [file behavsci-16-01654-s001.zip › behavsci-4448261-supplementary.pdf]

Supplementary Material S1

COVID-19 Preventive Health Behaviors, Parent-Child Conflict, and Depressive Symptoms among Chinese Adolescents

Table S1. Variables and estimated descriptive statistics

No.	Variable	Description	Observed N	M	SD
1	F_SES	Household socioeconomic status composite	1,938	0.006	0.728
2	E1	Preventive-practices item: attention to disease-prevention guidelines	2,543	4.552	0.738
3	E4	Preventive-practices item: mask use/other necessary preventive actions	2,543	4.245	0.847
4	E7	Preventive-practices item: increased handwashing	2,543	4.066	1.130
5	C1	Parent-child conflict item 1	2,543	2.001	1.158
6	C2	Parent-child conflict item 2	2,543	1.746	1.045
7	C3	Parent-child conflict item 3	2,543	1.833	1.110
8	C4	Parent-child conflict item 4	2,543	2.182	1.231
9	C5	Parent-child conflict item 5	2,543	2.210	1.236
10	C6	Parent-child conflict item 6	2,543	2.045	1.191
11	C7	Parent-child conflict item 7	2,543	1.976	1.189
12	D1	Depressive-symptom parcel 1: DEP4, DEP6, DEP11, DEP19, and DEP20	2,543	1.655	0.531
13	D2	Depressive-symptom parcel 2: DEP1, DEP3, DEP8, DEP13, and DEP18	2,543	1.593	0.568
14	D3	Depressive-symptom parcel 3: DEP2, DEP9, DEP10, DEP12, and DEP15	2,543	1.467	0.507
15	D4	Depressive-symptom parcel 4: DEP5, DEP7, DEP14, DEP16, and DEP17	2,543	1.536	0.547
16	GENDER	Gender (0 = boys, 1 = girls)	2,543	0.487	0.500
17	AGE	Age in years	2,543	13.067	1.561
18	COVLIVE	Community COVID-19 cases (0 = no, 1 = yes)	2,543	0.119	0.324
19	COVREL	COVID-19 exposure among relatives/close friends (0 = no, 1 = yes)	2,543	0.011	0.102

Note. Analytic N = 2,543. F_SES was observed for 1,938 participants and handled using the auxiliary distribution equation described in the Mplus syntax. Means and covariances are maximum-likelihood estimated sample statistics. D1-D4 are five-item mean parcels.

Estimated Covariance Matrix

Table S2, Panel A. Covariances involving Variables 1-10

Var.	1	2	3	4	5	6	7	8	9	10
1	0.529391									
2	0.038578	0.544613								
3	0.015711	0.360099	0.717610							
4	0.049006	0.201284	0.199379	1.276458						
5	-0.001116	-0.117618	-0.174003	-0.166784	1.341722					
6	-0.015187	-0.142016	-0.175671	-0.185467	0.929024	1.092963				
7	-0.026015	-0.135086	-0.181169	-0.189969	0.914013	0.895086	1.233178			
8	0.001867	-0.106524	-0.179459	-0.155069	1.048618	0.912773	0.934020	1.515559		
9	0.016170	-0.103269	-0.164779	-0.149063	0.926693	0.789399	0.846734	1.114426	1.527277	
10	0.010326	-0.146460	-0.209288	-0.199195	0.937833	0.845131	0.912788	1.030321	1.087633	1.417931
11	-0.033719	-0.113605	-0.177065	-0.176587	0.974458	0.929132	0.941790	1.048722	1.016358	1.084433
12	-0.015276	-0.079756	-0.091960	-0.061096	0.258234	0.247427	0.238746	0.267258	0.234167	0.268064
13	-0.022528	-0.096169	-0.115586	-0.070706	0.308853	0.305774	0.291136	0.316440	0.287071	0.318766
14	-0.026825	-0.097074	-0.105374	-0.077202	0.252405	0.255669	0.250771	0.255220	0.235009	0.265804
15	-0.022638	-0.103352	-0.123574	-0.085396	0.309842	0.296544	0.290966	0.312085	0.289901	0.319357
16	0.011970	0.017472	0.019652	0.050584	-0.011787	-0.010123	-0.009411	-0.014981	-0.009900	-0.029111
17	-0.244478	-0.075812	-0.067524	0.003475	0.152130	0.208069	0.154704	0.193124	0.131066	0.084275
18	0.009339	-0.005355	-0.013018	0.010683	0.034905	0.029335	0.031251	0.027186	0.028936	0.028841
19	0.001106	-0.004285	-0.002605	0.000482	0.002744	0.001513	0.000595	0.001217	0.004849	0.003845

Table S2, Panel B. Covariances among Variables 11-19

Var.	11	12	13	14	15	16	17	18	19
11	1.412735								
12	0.290874	0.282036							
13	0.344621	0.230609	0.323079						
14	0.287926	0.207228	0.232140	0.256844					
15	0.344073	0.219258	0.245270	0.219816	0.299237				
16	-0.019963	0.019026	0.011745	0.003407	0.005556	0.249837			
17	0.223756	0.095033	0.141808	0.103923	0.119791	-0.012122	2.435169		
18	0.034654	0.025883	0.028062	0.025907	0.025654	0.006630	0.000712	0.104654	
19	0.005363	0.002956	0.003379	0.003697	0.003429	0.000332	-0.000710	0.005817	0.010505

Note. The lower triangular covariance matrix is reported to six decimal places. Variable numbers correspond to Table S1. The matrix contains aggregate statistics only and no individual-level records.

Mplus Syntax for the Primary Model

```
TITLE:
    M2 FINAL Bootstrap-Only Latent Moderated Mediation with Child-Reported Conflict;
    Cluster bootstrap, FIML for missing family SES;

  DATA:
    FILE IS analysis_data_mplus.csv;

  VARIABLE:
    NAMES ARE
      gender age F_SES covlive covrel clusid
      e1 e4 e7
      c1 c2 c3 c4 c5 c6 c7
      p1 p2 p3 p4 p5 p6 p7 p8 p9 p10 p11 p12
      pc1 pc2 pc3
      DEP1 DEP2 DEP3 DEP4 DEP5 DEP6 DEP7 DEP8 DEP9 DEP10
      DEP11 DEP12 DEP13 DEP14 DEP15 DEP16 DEP17 DEP18
      DEP19 DEP20
      d1 d2 d3 d4
      pp cf_c cf_p dep_sum;

    USEVARIABLES ARE
      gender age F_SES covlive covrel
      e1 e4 e7
      c1 c2 c3 c4 c5 c6 c7
      d1 d2 d3 d4
      w;

    CLUSTER = clusid;
    WEIGHT = w;
    MISSING ARE ALL(-999);

  DEFINE:
    ! Constant unit weight required for bootstrap replicate weights
    ! with TYPE = COMPLEX in this Mplus installation.
    w = 1;

  ANALYSIS:
    TYPE = COMPLEX RANDOM;
    ESTIMATOR = ML;
    ALGORITHM = INTEGRATION;
    INTEGRATION = GAUSSHERMITE(7);
    REPSE = BOOTSTRAP;
    BOOTSTRAP = 5000;
    PROCESSORS = 4;

  MODEL:
    ! Measurement model
    PP BY
      e1
      e4
      e7;

    CONFL_C BY
      c1
      c2
      c3
      c4
      c5
      c6
      c7;

    DEP BY
      d1
      d2
      d3
      d4;

    ! Auxiliary distribution equation for missing family SES.
    ! This is used for FIML and is not a substantive directional path.
```

```
  F_SES ON
    PP
    gender
    age
    covlive
    covrel;

  ! Latent-by-gender interactions; gender: 0 = boys, 1 = girls
  XG | PP XWITH gender;
  MG | CONFL_C XWITH gender;

  ! Mediator equation
  CONFL_C ON
    PP          (a1)
    gender      (a2)
    XG          (a3)
    age         (am1)
    F_SES       (am2)
    covlive     (am3)
    covrel      (am4);

  ! Outcome equation
  DEP ON
    PP          (cp1)
    gender      (cp2)
    XG          (cp3)
    CONFL_C     (b1)
    MG          (b3)
    age         (ay1)
    F_SES       (ay2)
    covlive     (ay3)
    covrel      (ay4);

MODEL CONSTRAINT:
  NEW(
    ab ag da
    bb bg db
    cb cg dc
    ib ig dind
    tb tg dtot
  );

  ! Conditional a paths
  ab = a1;
  ag = a1 + a3;
  da = a3;

  ! Conditional b paths
  bb = b1;
  bg = b1 + b3;
  db = b3;

  ! Conditional direct associations
  cb = cp1;
  cg = cp1 + cp3;
  dc = cp3;

  ! Conditional indirect associations
  ib = a1*b1;
  ig = (a1+a3)*(b1+b3);
  dind = ig-ib;

  ! Conditional total associations
  tb = cb+ib;
  tg = cg+ig;
  dtot = tg-tb;

OUTPUT: SAMPSTAT TECH1 TECH8 TECH9 CINTERVAL(BOOTSTRAP);
```
